# Syndecan-1 is a novel molecular marker for triple negative inflammatory breast cancer and modulates the cancer stem cell phenotype via the IL-6/STAT3, Notch and EGFR signaling pathways

**DOI:** 10.1186/s12943-017-0621-z

**Published:** 2017-03-07

**Authors:** Sherif Abdelaziz Ibrahim, Ramy Gadalla, Eslam A. El-Ghonaimy, Omnia Samir, Hossam Taha Mohamed, Hebatallah Hassan, Burkhard Greve, Mohamed El-Shinawi, Mona Mostafa Mohamed, Martin Götte

**Affiliations:** 1 Department of Zoology, Faculty of Science, Cairo University, 12613 Giza, Egypt; 2 Department of Radiotherapy–Radiooncology, University Hospital Münster, Münster, Germany; 3 Department of General Surgery, Faculty of Medicine, Ain Shams University, 11566 Cairo, Egypt; 4 Department of Gynecology and Obstetrics, Münster University Hospital, Albert-Schweitzer-Campus 1, D11, 48149 Münster, Germany

**Keywords:** Inflammatory breast cancer, Syndecan-1, Proteoglycan, Cancer stem cell, IL-6/STAT3, Notch, EGFR

## Abstract

**Background:**

Inflammatory breast cancer (IBC), a particularly aggressive form of breast cancer, is characterized by cancer stem cell (CSC) phenotype. Due to a lack of targeted therapies, the identification of molecular markers of IBC is of major importance. The heparan sulfate proteoglycan Syndecan-1 acts as a coreceptor for growth factors and chemokines, modulating inflammation, tumor progression, and cancer stemness, thus it may emerge as a molecular marker for IBC.

**Methods:**

We characterized expression of Syndecan-1 and the CSC marker CD44, Notch-1 & -3 and EGFR in carcinoma tissues of triple negative IBC (*n* = 13) and non-IBC (*n* = 17) patients using qPCR and immunohistochemistry. Impact of siRNA-mediated Syndecan-1 knockdown on the CSC phenotype of the human triple negative IBC cell line SUM-149 and HER-2-overexpressing non-IBC SKBR3 cells employing qPCR, flow cytometry, Western blotting, secretome profiling and Notch pharmacological inhibition experiments. Data were statistically analyzed using Student’s t-test/Mann-Whitney U-test or one-way ANOVA followed by Tukey’s multiple comparison tests.

**Results:**

Our data indicate upregulation and a significant positive correlation of Syndecan-1 with CD44 protein, and Notch-1 & -3 and EGFR mRNA in IBC vs non-IBC. ALDH1 activity and the CD44^(+)^CD24^(-/low)^ subset as readout of a CSC phenotype were reduced upon Syndecan-1 knockdown. Functionally, Syndecan-1 silencing significantly reduced 3D spheroid and colony formation. Intriguingly, qPCR results indicate downregulation of the IL-6, IL-8, CCL20, gp130 and EGFR mRNA upon Syndecan-1 suppression in both cell lines. Moreover, Syndecan-1 silencing significantly downregulated Notch-1, -3, -4 and Hey-1 in SUM-149 cells, and downregulated only Notch-3 and Gli-1 mRNA in SKBR3 cells. Secretome profiling unveiled reduced IL-6, IL-8, GRO-alpha and GRO a/b/g cytokines in conditioned media of Syndecan-1 knockdown SUM-149 cells compared to controls. The constitutively activated STAT3 and NFκB, and expression of gp130, Notch-1 & -2, and EGFR proteins were suppressed upon Syndecan-1 ablation. Mechanistically, gamma-secretase inhibition experiments suggested that Syndecan-1 may regulate the expression of IL-6, IL-8, gp130, Hey-1, EGFR and p-Akt via Notch signaling.

**Conclusions:**

Syndecan-1 acts as a novel tissue biomarker and a modulator of CSC phenotype of triple negative IBC via the IL-6/STAT3, Notch and EGFR signaling pathways, thus emerging as a promising therapeutic target for IBC.

**Electronic supplementary material:**

The online version of this article (doi:10.1186/s12943-017-0621-z) contains supplementary material, which is available to authorized users.

## Background

Inflammatory breast cancer (IBC), the most aggressive form of breast cancer, represents approximately 2.5% of newly diagnosed breast cancers in the United States [1]. This percentage reaches an even higher level of 5–10% of breast cancer cases in North African countries such as Tunisia, Morocco, and Egypt [2, 3]. IBC is a unique disease characterized by erythema, edema of the breast, a “peau d’orange” and formation of lymphatic tumor emboli [4–6]. IBC patients have a poor survival rate with a median of 3 years compared with non-IBC [1] with no currently available targeted therapies. Based on the surrogate markers estrogen receptor or progesterone receptor (ER/PR) status and human epidermal growth factor receptor (HER)-2 expression, breast cancer can be classified into ER^+^ (ER^+^/PR^+^ and HER-2^−^), ER^+^HER-2^+^ (ER^+^/PR^+^ and HER-2^+^), HER-2^+^ (ER^−^/PR^−^ and HER-2^+^), and triple negative (ER^−^/PR^−^ and HER-2^−^) [7, 8]. IBC possess the same molecular subtypes as non-IBC [9, 10], with more than 50% being reported as ER^−^, 36–60% HER-2^+^, and 30% triple negative according to a multinational IBC registry [1, 4]. Therefore, the percentage of triple negative breast cancer is higher for IBC compared to non-IBC cases [7, 11, 12]. Several lines of evidence indicate that the aggressive phenotype of IBC is due to enrichment for chemo- and radioresistant cancer stem cells (CSCs) [13]. These cells are characterized by self-renewal, unlimited and high proliferative potential, expression of multidrug-resistance proteins, efficient DNA repair capacity and apoptosis resistance [14, 15]. Using flow cytometry, CSCs can be distinguished from the bulk of the tumor by their expression of cell surface makers CD44 and CD24 (as a CD44^(+)^CD24^(-/low)^ subpopulation) and based on the activity of ALDH1 [16]. Due to their functional link to therapeutic resistance, CSCs represent an attractive therapeutic target to dampen tumor recurrence [15, 16].

Syndecan-1 (CD138), a cell surface heparan sulfate proteoglycan, emerges as a candidate target for IBC. It acts as a coreceptor for a multitude of biological factors like growth factors, angiogenic factors, cytokines and chemokines [17–21]. Dysregulated expression and a potential role of Syndecan-1 as a modulator of cell proliferation and invasive growth have been demonstrated in different tumor entities including breast cancer [22–26]. The function and (de)differentiation state of CSCs are substantially modulated by many interconnected signaling pathways e.g. IL-6/STAT3, Hedgehog, WNT and Notch signaling that emerge as relevant therapeutic targets [27, 28]. Interestingly, we and others uncovered the regulatory role played by Syndecan-1 in IL-6/STAT3 and WNT signaling in the human triple negative (MDA-MB-231) and hormone-receptor positive (MCF-7) non-IBC cell lines [16], and in Syndecan-1- knockout mice [29, 30]. While these data suggest that a therapeutic targeting of Syndecan-1 may be a mean of synchronously interfering with multiple relevant pathogenetic routes, the precise role of Syndecan-1 in modulating IBC pathogenesis and its CSC phenotype is still unexplored.

The cell surface epidermal growth factor receptor (EGFR) is overexpressed in approximately 50% of triple negative IBC [31]. Patients with EGFR-positive tumors are characterized by lower survival rates and are associated with the risk of higher tumor recurrence [32, 33]. EGFR and/or HER-2 overexpression, and MAPK hyperactivation lead to activation of NFκB associated with ER downregulation in IBC specimens [34]. Moreover, a significantly positive correlation between EGFR and CD44 expressions exists in breast invasive ductal carcinoma patients and that is associated with the worst prognosis [35]. Interestingly, in a study of 230 surgical specimens of primary colorectal carcinoma, epithelial positive Syndecan-1 immunostaining was significantly associated with tumor size and EGFR expression [36].

In this study, we examined the expression of Syndecan-1 and its correlation with the CSC marker CD44, Notch-1 & -3 and EGFR expression in carcinoma tissues of triple negative IBC and non-IBC patients. We further employed siRNA-mediated Syndecan-1 knockdown in the human IBC cell line SUM-149 and HER-2 overexpressing non-IBC SKBR3 cells to decipher its impact on a CSC phenotype (CD44^(+)^CD24^(-/low)^ and ALDH1^+^ subpopulations). Of particular importance,, we studied the expression and activity of several distinct signaling pathways relevant for CSC function to address possible underlying molecular mechanism(s) for this effect. Supported by an unbiased cytokine array screening approach, we specifically tested the effect of Syndecan-1 depletion on inflammatory signaling, including the IL-6/STAT3 signaling pathway [37–39]. Furthermore, we investigated a potential impact on the stemness-associated Notch and EGFR pathways [35, 39]. Our data demonstrate that Syndecan-1 expression is higher in tissues of triple negative IBC than that in non-IBC. Further, Syndecan-1 is a modulator of the CSC phenotype of IBC via IL-6/STAT-3, Notch and EGFR signaling. Therefore, Syndecan-1 may act as a novel marker for this disease and its targeting could have therapeutic implications for IBC patients.

## Methods

### Antibodies and reagents

The antibodies against p-STAT3^(Y705)^, STAT-3, p-NFκB-p65^(Ser276)^, NFκB-p65, p-Akt^(Ser473)^, Akt and CD44 (clone 156-3-c11) were from Cell Signaling Technology, Inc. (Beverly, MA, USA), gp130 antibody was purchased from R&D Systems (Minneapolis, MN, USA). Anti-human Notch-1 and EGFR antibodies were from Santa Cruz Biotechnology (Santa Cruz, CA, USA), anti-human-CD44-FITC, anti-human-CD24-PE, IgG2b-FITC, IgG1-PE antibodies and rhEGF were obtained from Immunotools (Friesoythe, Germany), and anti-Syndecan-1 (clone B-A38) was from Biorad (Hercules, CA, USA). Anti-human-Notch-2-PE & APC, Syndecan-1 (CD138)-PE antibodies were from eBioscience, Inc. (San Diego, CA, USA) and HRP–conjugated secondary antibodies were from KPL (Gaitherburg, MD, USA). Gamma-secretase inhibitor (GSI) was from Calbiochem (Darmstadt, Germany). Media, fetal calf serum (FCS) and tissue culture supplies were from Lonza (Basel, Switzerland). Unless otherwise stated, all chemicals were from Sigma (St. Louis, MO, USA).

### Cell culture

The human IBC cell line SUM-149 (a kind gift from Dr. Bonnie Sloane, Wayne State University, Detroit, MI, USA) and the non-IBC cell line SKBR3 (ATCC/LGC Promochem, Wesel, Germany) were maintained in HAM’s-F12 and DMEM containing 10% FCS, 1% glutamine and 1% penicillin/streptomycin in a humidified atmosphere of 5% CO_2_ at 37 °C, respectively.

### Patient’s samples

We enrolled 30 triple negative breast cancer patients from the breast clinic of Ain Shams university hospitals (IBC *n* = 13, non-IBC *n* = 17). Carcinoma tissues were divided into two parts: one part was fixed in 10% neutral formalin buffered for immunohistochemical staining and the other part was frozen in -80 °C for subsequent isolation of total RNA.

**Table 1:** Clinical and pathological data of IBC and non-IBC patients

Characteristic	IBC (*N* = 13)	Non-IBC (*N* = 17)	*P* value
Age			
Range	29–60	35–63	0.119^a^
Mean ± SD	45.15 ± 8.98	50.11 ± 9.43	
NA	0	0	
Tumor size			
≤ 4	2 (16.7%)	2 (11.8%)	1.000^b^
> 4	10 (83.3%)	15 (88.2%)	
NA	1	0	
Lymph node status			
< 4	1 (10%)	7 (41.2%)	0.098^b^
≥ 4	9 (90%)	10 (58.8%)	
NA	3	0	
Tumor grade			
G1	0	0	0.332^b^
G2	7 (58.3%)	14 (82.4%)	
G3	4 (33.3%)	3 (17.6%)	
G4	1 (8.3%)	0	
NA	1	0	
Lymphovascular invasion			
Negative	3 (27.3%)	13 (76.5%)	0.018*^b^
Positive	8 (72.7%)	4 (23.5%)	
NA	2	0	

Data are expressed as mean ± SD

*NA* Data not available

*significant *P* value calculated by ^a^Student’s t-test or ^b^Fisher’s exact test

### Immunohistochemical staining of CD44 and Syndecan-1

Immunohistochemical staining was performed on serial formalin-fixed and paraffin- embedded tissues sectioned at 4 μm-thickness as we previously described [40]. Tissue sections were deparaffinized by two consecutive incubations in xylene for 10 min each, followed by rehydration through two changes of absolute ethanol, graded decreasing concentrations of ethanol for 5 min each and finally in distilled water. For antigen retrieval, slides were incubated in citrate buffer (pH = 6.0) in a water steamer for 30 min. Slides were left to cool at room temperature for 20 min then washed 3 × 5 min with PBS. Endogenous peroxidase activity of the tissue was blocked with 3% hydrogen peroxide for 5 min (Dual Endogenous Enzyme block, Dako K4065, Glostrup, Denmark) and slides were washed with PBS 3 × 5 min. Tissue sections were blocked in 1% BSA/PBS and incubated overnight at 4 °C in a humidified chamber with the primary anti-CD44 (dilution 1:800) and anti-Syndecan-1 antibodies (dilution 1:100). Afterwards, slides were washed 3 × 5 min and incubated with HRP-Rabbit/Mouse (DAKO EnVision + Dual Link System-HRP (DAB+) for 30 min at room temperature. Then, nuclei were counterstained with hematoxylin, sections were mounted with Permount® and imaged. Negative control slides were run in parallel where primary antibodies were omitted.

### siRNA-mediated knockdown of Syndecan-1 expression

siRNA knockdown was performed using a negative control siRNA (negative control #1, Ambion, Cambridgeshire, UK) and siRNA #12634 (Ambion) to target Syndecan-1 coding region. Cancer cell lines were transfected with 20 nM siRNA using Dharmafect reagent (Dharmacon, Lafayette, CO, USA) according to the manufacturer’s instructions. Successful knockdown was confirmed by flow cytometry as previously described [16, 22].

### Flow cytometry

To detect cell surface breast CSC markers, control and Syndecan-1 siRNA transfected cells were incubated with 10 μl of anti-CD44-FITC, anti-CD24-PE and the FITC and PE isotype control antibodies for 30 min at room temperature in the dark. Analogously, cells were analyzed for Syndecan-1 (CD138)-PE in combination with Notch-2-PE or-APC antibodies. Stained cells were analyzed by a cube-8 flow cytometer (Sysmex/Partec, Muenster, Germany). For ALDH1 activity assessment, 1× 10^6^ control and Syndecan-1 siRNA transfected cells were resuspended in assay buffer containing ALDH1 substrate (1 μmol/L). Half of this suspension was incubated with 50 mM ALDH1 inhibitor diethylaminobenzaldehyde (DEAB) as negative control. Afterwards, the cells were incubated for 1 h at 37 °C in water bath in dark with agitation at 10 min interval. Finally, the cells were centrifuged at 400 xg for 5 min and were resuspended in 1 mL assay buffer and stored on ice prior to acquisition by flow cytometry.

### Quantitative real-time PCR

Total RNA isolated from cultured cells or frozen tissues using GeneJET RNA Purification Kit (Thermoscientific, Waltham, USA) was reverse transcribed into cDNA using the high capacity cDNA Kit (Applied Biosystems, Foster City, CA, USA). Quantitative real-time PCR was conducted in duplicate for each gene of interest using SYBR Green dye and gene expression levels were measured in a steponeplus detection System (Applied Biosystems). Relative gene expression was evaluated using the 2^-∆∆^Ct method after normalization to 18S rRNA or GAPDH as previously described [22]. Melting curve analysis was performed to confirm specific product amplification. Primers were designed using Primer 3.0 software or referred to the published literature. Primer sequences are listed in Additional file 1: Table S1. For Notch pharmacological inhibition experiments, 1 μM GSI was added for control and Syndecan-1 siRNA transfected SUM-149 cells 24 h before RNA extraction. Data for mRNA expression levels in carcinoma tissues of IBC vs non-IBC (normalized to values of normal tissues collected during reduction mammoplasty) was represented as log2-transformed fold change.

### Sodium dodecyl sulfate-polyacrylamide gel electrophoresis (SDS-PAGE) and immunoblotting

Briefly, 72 h post transfection, control and Syndecan-1 siRNA transfected cells were washed twice with PBS and lysed in RIPA buffer containing protease and phosphatase inhibitors [22]. The cell lysates were shaked for 20 min followed by centrifugation at 10,000 x*g* for 10 min at 4 °C. Supernatant was collected and protein concentration was determined using Bradford assay (Fermentas, Burlington, ON, Canada). 25–50 μg of protein per lane was separated on 10–12% gels and electrotransferred into polyvinylidene fluoride (PVDF) membrane (Millipore, USA). Immunoblotting was performed using primary antibodies against phospho-NFκB/p65^(Ser276)^, phospho-STAT3^(Y705)^, phospho-Akt^(Ser473)^, Akt, gp130, Notch-1, EGFR and HRP–conjugated secondary antibodies. After washing, specifically bound antibodies were visualized by ECL reaction. Visualized bands were analyzed with ImageJ software (National Institutes of Health, Bethesda, MA, USA) using β-actin or tubulin as loading controls.

### Three dimensional (3D) spheroids and colony formation assays

Petri-dishes were coated with 150 μl Cultrex®Basement Membrane Extract (BME) (Trevigen, Inc., MD, USA) and incubated at 37 °C in a CO_2_ incubator for 15 min to solidify. Control and Syndecan-1 siRNA transfected SUM-149 and SKBR3 cells were mixed with 2% BME at density of 5 × 10^4^ before overlaying onto each coated petridish and incubated for 7–10 days at 37 °C to allow spheroid formation in 3D. The media were changed every 3–4 days, the spheroids were stained with cell tracker red dye, and the number of spheroids (**>**50 μm) was counted. To examine the effect of Syndecan-1 silencing on clonogenic ability, 10,000 control and Syndecan-1 knockdown SUM-149 cells were seeded in six-well plates and maintained in Ham-F12 with 10% FBS for 10–14 days as previously performed [41]. Cells were washed with PBS, fixed in methanol for 20 min and stained with 0.05% crystal violet for 15 min. Excess stain was removed by water and the stain was dissolved in 1 ml 10% glacial acetic acid. The released color was measured by spectrophotometry at 595 nm according to [42]. Colony formation steps were also performed in presence of 10 ng/mL EGF and 1% FBS (with addition of fresh media at interval 3–4 days) or 1 μM GSI for 24 h followed by exchange with complete growth media.

### Secretome profiling of conditioned media of SUM-149 cells grown in 3D spheroids

Cytokines, chemokines and growth factors secreted by control and Syndecan-1-silenced SUM-149 cells grown in 3D were detected in conditioned media (CM) using RayBio cytokine array-C3 (RayBiotech, Inc. GA, USA). All steps needed to form 3D spheroids were analogously performed followed by starvation for 24 h. Media conditioned by the secretome of the cells were collected and subjected to profile 42 biological factors according to the manufacturer’s instructions. The signal intensity of each spot, which represents the secreted chemokine, cytokines, and growth factors was evaluated by subtracting from the background and normalized to positive controls using ImageJ software as we previously described [40].

### Statistical analysis

All Data are presented as mean ± SEM or SD as indicated. Differences among variables were evaluated using χ^2^, or Fischer’s exact tests. Student’s t-test (for normally distributed data) or Mann-Whitney U-test (for non-normally distributed data) was used for two group comparisons. The statistical difference between more than two groups was evaluated by one-way ANOVA followed by Tukey’s multiple comparison test. The Pearson’s Rank correlation test was used to analyze the correlations. The level of significance was set at *p* < 0.05. Graphs were plotted and analyses were performed by GraphPad Prism 7 software (San Diego, CA, USA) and IBM SPSS version 22 (Chicago, IL, USA).

## Results

### Clinical and pathological characteristics of patients

The clinical and pathological characteristics of patients included in this study are represented in Table 1. There were 13 IBC patients with an average age of 45.15 years (range from 29 to 60 years) and 17 non-IBC patients with an average age of 50 years (range from 35 to 63 years). In the IBC group 16.7% of the patients had a tumor size ≤ 4 and 83.3% of the patients had a tumor size > 4, while in the non-IBC group 11.8% of the patients had a tumor size ≤ 4 and 88.2% of the patients had a tumor size > 4. The histological tumor grade was diagnosed as: 58.3% grade 2 (G2), 33.3% grade 3 (G3) and 8.3% was Grade 4 (G4) in IBC and was diagnosed as: 82.4% grade 2 (G2) and 17.6% grade 3 (G3) in non-IBC. The lymph nodes metastasis status was subdivided according to the number of positive metastatic lymph nodes into <4 and ≥ 4. All IBC patients who underwent surgery were lymph nodes metastasis positive: 10% had <4 positive lymph nodes and 90% had ≥ 4 positive metastatic lymph nodes. In non-IBC patients, 41.2% had < 4 lymph nodes involvement and 58.8% had ≥ 4 positive lymph nodes. Therefore, there is a trend toward women with IBC showing increased incidence of ≥ 4 positive metastatic lymph nodes compared with non-IBC women (*P* = 0.098). Pathological examination of IBC and non-IBC tissue sections revealed that lymphovascular invasion is positive in 72.7% and 23.5% in IBC and non-IBC, respectively. The presence of lymphovascular invasion in carcinoma tissues of IBC was significantly higher (*P* = 0.018) than that in non-IBC.

### Higher expression with a positive correlation of Syndecan-1 with CD44 in carcinoma tissues of triple-negative IBC vs non-IBC patients

**Fig. 1:**
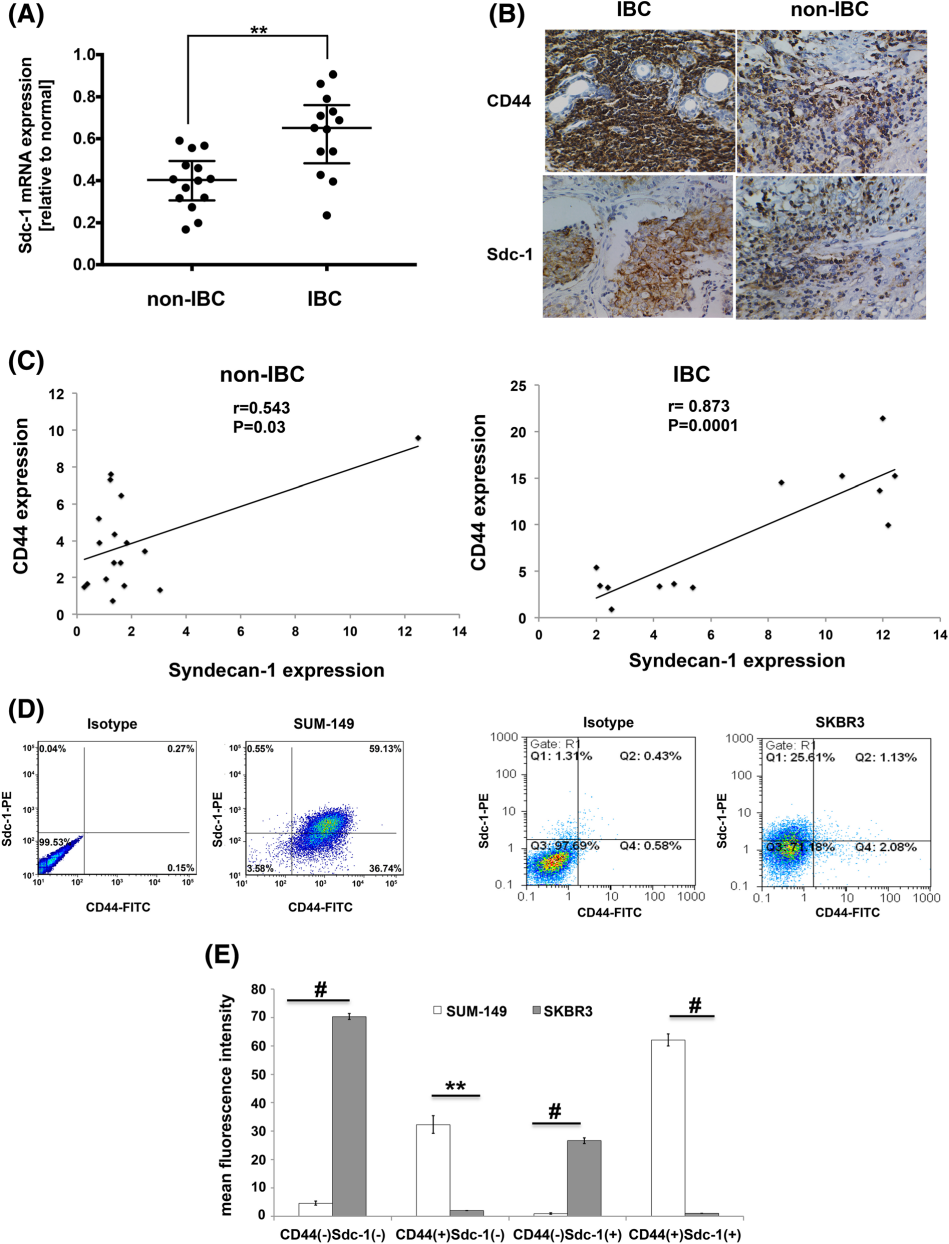
Expression of Syndecan-1 and the CSC marker CD44 in carcinoma tissues of IBC vs non-IBC patients, SUM-149 and SKBR3 cells. **a** Higher expression of Syndecan-1 mRNA level in carcinoma tissues of IBC (*n* = 13) vs non-IBC (*n* = 14). RQ values of mRNA expression are log2-transformed and normalized to values of normal tissues collected during reduction mammoplasty. Bars represent median with interquartile range. ** *P* < 0.01 as determined by Mann-Whitney U-test. **b** Representative fields of immunostaining (*brown color*) of Syndecan-1 and CD44 in paraffin embedded carcinoma tissue sections of triple negative IBC (*n* = 13) and non-IBC (*n* = 17) patients. A high density of cancer cells positive for CD44 and Syndecan-1 is observed in IBC vs non-IBC. **c** Pearson’s correlation between Syndecan-1 and CD44 expression in carcinoma tissues of IBC vs non-IBC. **d** A representative flow cytometric analysis for the expression of CD44 and Syndecan-1 in SUM-149 and SKBR3 cells. **e** Quantitative analysis of four subpopulations; CD44^(-)^Syndecan-1^(-)^, CD44^(+)^Syndecan-1^(-)^, CD44^(-)^Syndecan-1^(+)^ and CD44^(+)^Syndecan-1^(+)^. Syndecan-1 is higher expressed in the CD44^(+)^-enriched subset in SUM-149 cells than that in SKBR3 cells. Data represent mean ± SEM, n ≥ 3. ** *P* < 0.01, # *P* < 0.001 as determined by Student’s t-test. Data shown are a single experiment representative of three independent experiments

Although Syndecan-1 expression is a prognostic marker for different tumor entities including breast cancer, and is a modulator of breast and prostate CSCs [16, 43], its role in IBC pathogenesis is still unknown. Therefore, we analyzed Syndecan-1 expression by qPCR or immunohistochemical staining in carcinoma tissues of triple negative IBC vs non-IBC patients. Relative to non-IBC, our data indicate a significantly higher expression of Syndecan-1 transcript levels (*P* < 0.01) (Fig. 1a), and higher positive staining of Syndecan-1 protein in tissues of IBC (*P* < 0.01) (Fig. 1b), and on carcinoma cells infiltrated into lymphatic vessels, a unique feature for IBC (Additional file 2: Figure S1).

IBC is essentially characterized by chemo- and radio-resistance, which may be attributed to the existence of CSCs [13, 44]. Therefore, we next investigated expression of the CSC marker CD44 in triple negative IBC vs non-IBC. Our data showed that tissues of triple negative IBC exhibited a significantly higher CD44 staining than those of non-IBC patients (*P* < 0.05) (Fig. 1b). This finding suggests that IBC tissues may display higher CSC properties than those of non-IBC patients. Interestingly, a significant positive correlation was found between expression of Syndecan-1 and CD44 in IBC (*r* = 0.87, *P* < 0.001) and in non-IBC (*r* = 0.54, *P* < 0.05) (Fig. 1c), suggesting a functional association and an essential role in IBC patients.

We next investigated expression and distribution of Syndecan-1 and the CSC marker CD44 in our experimental models; the human triple negative IBC SUM-149 cell line and the HER-2 overexpressing non-IBC SKBR3 cell line. Our findings indicate that the CD44^(+)^Syndecan-1^(+)^ subset represents approximately 62.12% and 1.07% in SUM-149 and SKBR3 cells, respectively. The CD44^(+)^Syndecan-1^(-)^ subset represents 32.31% and 2.01%, and the CD44^(-)^Syndecan-1^(+)^ subset represents 0.98% and 26.57% in SUM-149 and SKBR-3 cells, respectively (Fig. 1d&e ). This means that the CD44^(+)^-enriched Syndecan-1 subset constitutes 98.5% and 3.9% of total Syndecan-1 expression in SUM-149 and SKBR3 cells, respectively. This conforms to our findings in the clinical tissue specimens and proves that Syndecan-1 is coexpressed and may possess a functional link to the CSC marker CD44 in triple negative IBC.

### Syndecan-1 silencing significantly reduces the CD44^(+)^CD24^(-/low)^ pool and ALDH1 activity in SUM-149 and SKBR3 cells

**Fig. 2:**
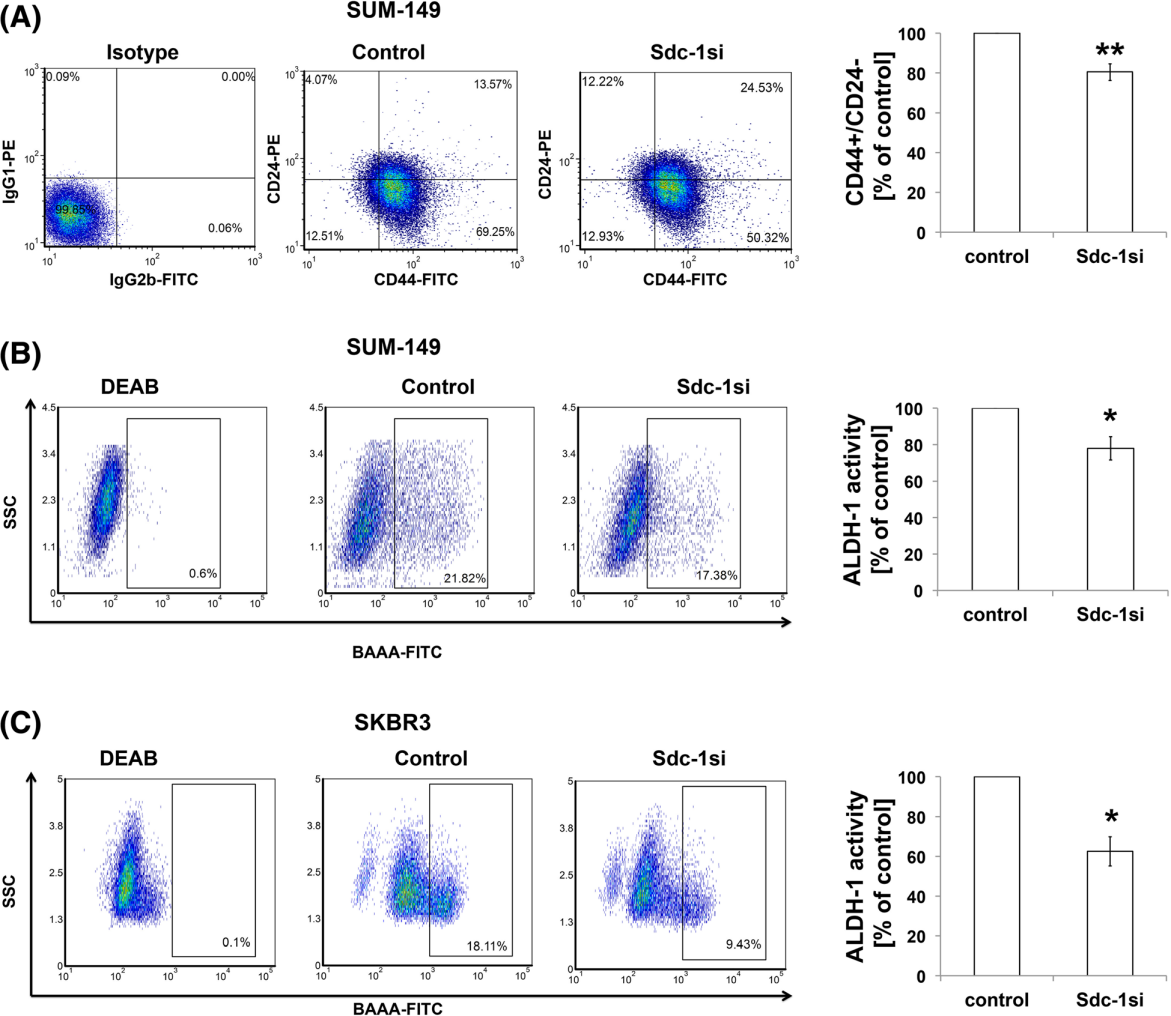
Syndecan-1 silencing reduces the CD44^(+)^CD24^(-/low)^ subpopulation and ALDH-1 activity in IBC SUM-149 and non-IBC SKBR-3 cells. **a** Left panel: A representative flow cytometric analysis of the stem cell-associated cell surface markers CD44 and CD24 in control and Syndecan-1-silenced SUM-149 cells. **b** and **c** Representative flow cytometric analysis of ALDH-1-positive SUM-149 and SKBR-3 cells, upon Syndecan-1 knockdown, respectively. Data shown are a single experiment representative of three independent experiments. Right panels of A-C show the quantification of CSC markers as analyzed by flow cytometry. Data represent mean ± SEM, n ≥ 3. ** *P* < 0.01, * *P* < 0.05 as determined by Student’s t-test

We have previously shown that Syndecan-1 is a modulator of breast cancer stemness in MDA-MB-231 and MCF-7 cells [16]. To formally test if Syndecan-1 is also of relevance for CSCs of SUM-149 and SKBR3 cells, we analyzed the effect of Syndecan-1 knockdown on CSC properties, namely the CD44^(+)^CD24^(-/low)^ subpopulation and ALDH1 activity. Successful downregulation of Syndecan-1 in both cell lines was confirmed by flow cytometry (Additional file 3: Figure S2). We next analyzed the expression of CD44 and CD24 in control and Syndecan-1-silenced SUM-149 cells by flow cytometry. siRNA-mediated Syndecan-1 depletion significantly reduced the CD44^(+)^CD24^(-/low)^ pool by 19.5% as compared with control cells (with an average of 66.2% ± 2.1% in control cells and 53.1% ± 1% in Syndecan-1 siRNA transfected cells) (*P* < 0.01, *n* = 4) (Fig. 2a). Although the CD44^(+)^CD24^(+)^ subset increased upon Syndecan-1 depletion, it did not reach the significance level (Fig. 2a).

We further characterized the activity of ALDH isoform 1 (ALDH1), an additional surrogate marker for CSCs [14] using Aldefluor assay. Flow cytometric analysis of ALDH1 activity uncovered that siRNA-mediated Syndecan-1 depletion diminished the ALDH1-positive subpopulation by 22% in SUM-149 cells (with an average of 24.6% ± 1.9% in control cells and 17.3% ± 1.1% in Syndecan-1siRNA cells, *P* < 0.05) (Fig. 2b) and by 42% in SKBR-3 cells (with an average of 18.9% ± 0.6% in control cells and 11.9% ± 1.8% in Syndecan-1 siRNA transfected cells, *P* < 0.01) (Fig. 2c) compared with control cells. Taken together, these findings further validate the key role played by Syndecan-1 in regulating the stem cell phenotype in different molecular subtypes of IBC and non-IBC cell lines.

### Syndecan-1 knockdown perturbs the formation of colonies and spheroids growing in 3D

**Fig. 3:**
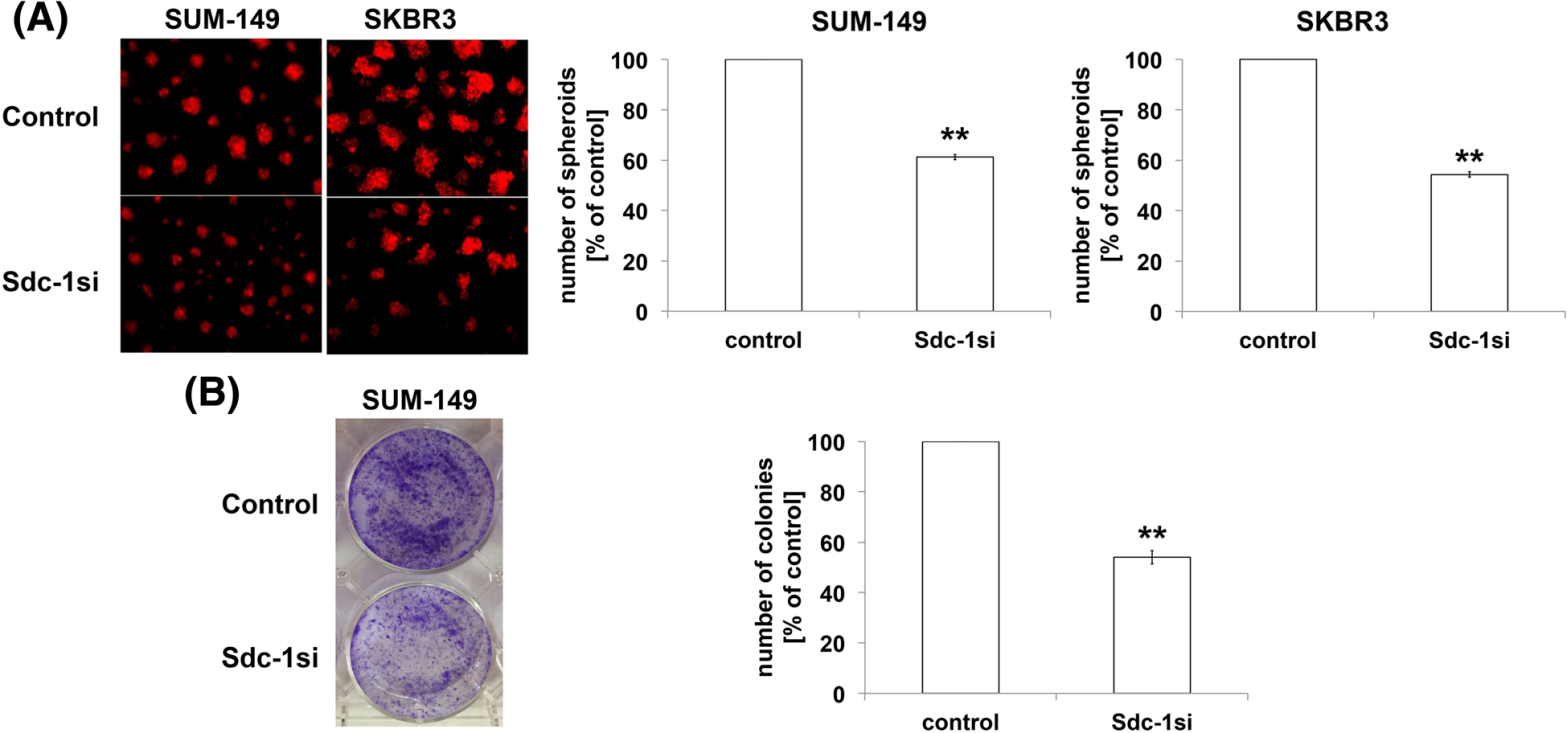
Syndecan-1 silencing impairs the ability of 3D spheroids and colony formation in IBC SUM-149 and non-IBC SKBR-3 cells. Equal numbers of control and Syndecan-1-silenced SUM-149 and SKBR-3 cells were either plated on cultrex-coated petri dishes to form spheroids in 3D (**a**) or plated on 2D to form colonies (**b**). The right panels of A and B represent the quantification of the size of spheroids or the number of colonies as indicated. Data represent mean ± SEM, *n* = 3. ** *P* < 0.01 as determined by Student’s t-test

Since colony and spheroids formation are unique properties for tumorigenesis and self-renewal of CSCs [16], we evaluated the influence of Syndecan-1 on this process in breast cancer cell lines in vitro. Single cell suspensions of control and Syndecan-1 siRNA transfected SUM-149 and SKBR3 cells in 2% cultrex were overlaid on cultrex-coated Petri-dishes and cultured for 7–10 days. Our data indicate that Syndecan-1-silenced cells displayed a significantly reduced capability to form spheroids in 3D by 39% and 46% in SUM-149 and SKBR3 cells compared to control cells, respectively (*P* < 0.01, *n* = 3) (Fig. 3a). We next examined the potential function of Syndecan-1 in regulating colony formation. Syndecan-1 ablation suppressed the colony forming capacity by 46% as compared to control SUM-149 cells (*P* < 0.01, *n* = 3) (Fig. 3b).

### Syndecan-1 silencing downregulates a myriad of cancer stem cell-related genes in SUM-149 and SKBR3 cells

**Fig. 4:**
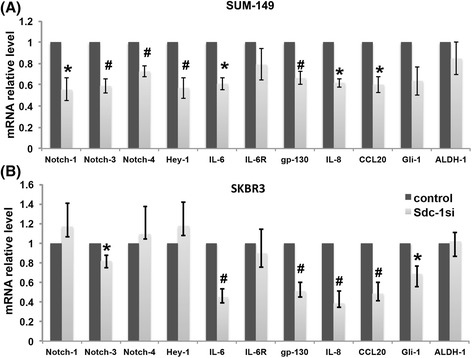
Syndecan-1 silencing suppresses CSC-related gene expression in SUM-149 and SKBR3 cells. Post Syndecan-1 knockdown, total RNA isolated from SUM-149 (**a**) and SKBR3 cells (**b**) was reverse transcribed into cDNA and subjected into qPCR. Data represent mean ± SEM, n ≥ 3. * *P* < 0.05, # *P* < 0.01 as determined by Student’s t-test

CSCs are regulated by several distinct signaling pathways, including the Notch, IL-6/STAT3, and hedgehog signaling pathways [28]. Therefore, we examined whether the mRNA expression levels of components of these pathways were influenced by Syndecan-1 depletion. As depicted in Fig. 4a&b, our qPCR data indicate that Syndecan-1 knockdown led to a significant downregulation of Notch-1, -3, -4 and the Notch signaling downstream target Hey-1 transcript levels by 45%, 41%, 27% and 43% in SUM-149 cells, respectively. In contrast, only a significantly reduction of Notch-3 transcript levels by 19% was evident in SKBR3 cells as compared to control cells. The activation of the Hedgehog pathway is mediated by the transcription factor Gli-1 [45]. Our data uncovered Gli-1 that transcript levels were downregulated by 31% in Syndecan-1 knockdown cells compared to control SKBR3 cells. This conforms with the observation that CD138/Syndecan-1^(+)^ multiple myeloma cells express Hedgehog genes and that inhibition of Smoothened decreased multiple myeloma cell viability by downregulating Gli-1 and Patched1 [46]. The expression of stemness-associated inflammatory cytokines namely; IL-6 and IL-8, and gp130 mRNAs were downregulated by 39%, 38% and 34% in Syndecan-1-silenced SUM149 and by 55%, 61% and 49% in SKBR3 upon Syndecan-1 knockdown, respectively. A relevant clue for regulation of IL-6 is the chemokine CCL20, which induces proliferation of cultured human breast epithelial cells [47] and is involved in IL-6 induction [48]. CCL20 mRNA was downregulated by 40% and 51% in Syndecan-1 knockdown SUM-149 and SKBR3 compared to control cells, respectively, suggesting the existence of a Syndecan-1/CCL20/IL-6 axis.

### Syndecan-1 siRNA knockdown reduces the secretome profile of SUM-149 cells

**Fig. 5:**
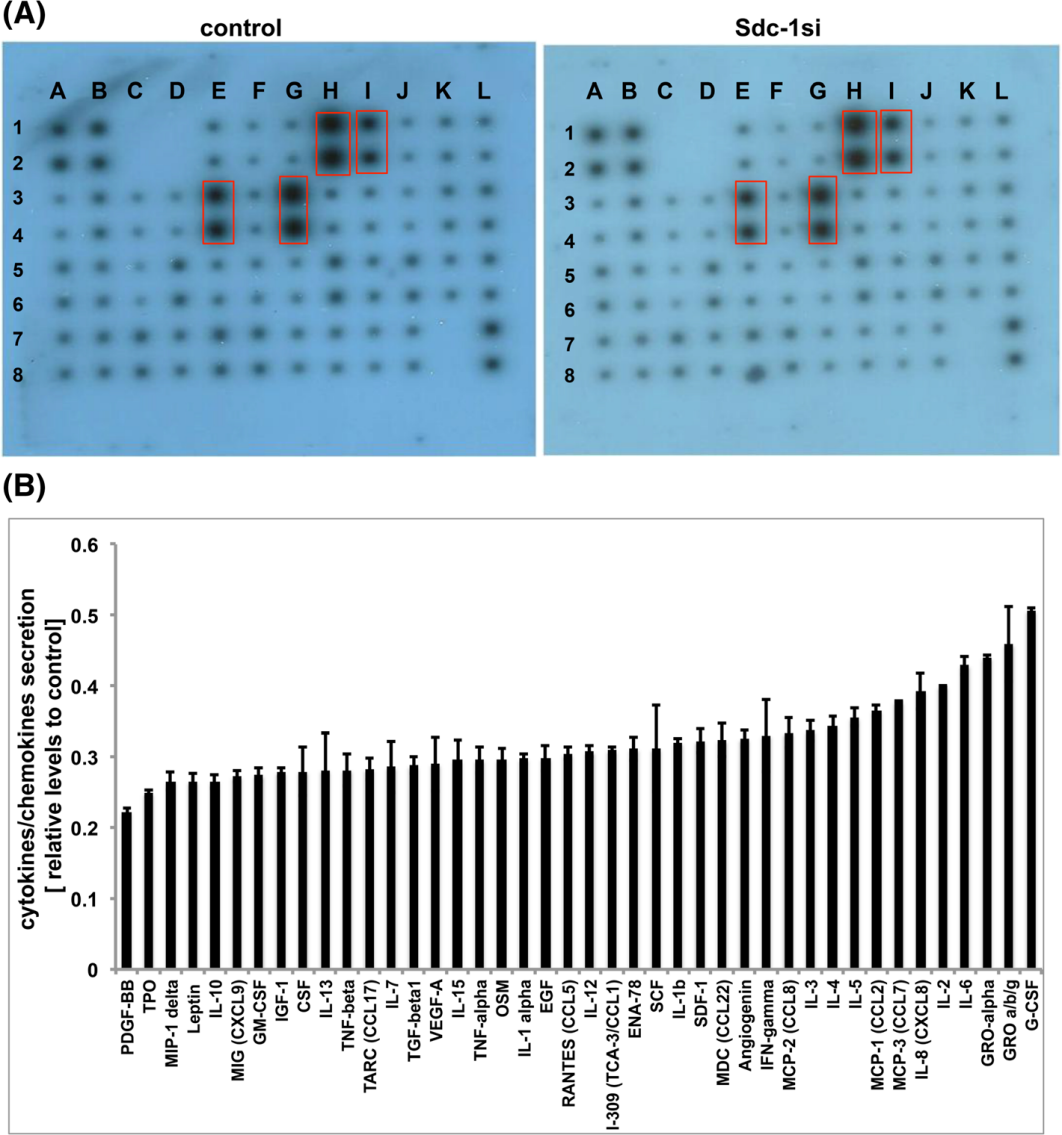
Profiling of cytokines/chemokines and growth factors secreted by control and Syndecan-1 siRNA transfected SUM-149 cells. Media conditioned by secretome of control and Syndecan-1-depleted cells for 24 h were subjected to cytokine profiling using RayBio cytokine array-3, which detects 42 different cytokines. **a** A representative picture of the human cytokine protein membrane array for the secretions of control and Syndecan-1 siRNA transfectants. **b** Densitometric quantification of the signal intensity of each cytokine secreted by the cells. Control values were set to 1. Red boxes represent the predominant secreted cytokines namely, IL-6 (E3,4), IL-8 (G3,4), GRO (H1,2) and GRO-α (I1,2)

We further analyzed the effect of Syndecan-1 silencing on the secretome profile of SUM-149 cells. This cell line is characterized by high secretion of IL-6 and IL-8 [41], which could promote a CSC phenotype via an autocrine feedback loop. Therefore, post starvation for 24 h, serum-free culture media collected from control and Syndecan-1-silenced SUM-149 cells were subjected to cytokine profiling. Densitometric analysis assessed by ImageJ software indicates an overall decrease in the secretions of cytokines, chemokines and growth factors by approximately 50–80% upon Syndecan-1 knockdown in SUM-149 cells (Fig. 5). Strikingly, the predominant cytokines, chemokines and growth factors secreted by SUM-149 cells implicated in regulating a CSC phenotype were downregulated; namely IL-6, IL-8 and growth regulated protein GRO-alpha, and GRO a/b/g.

### Syndecan-1 silencing downregulates gp130 and attenuates the constitutive activity of STAT3 and NFκB in SUM-149 and SKBR3 cells

**Fig. 6:**
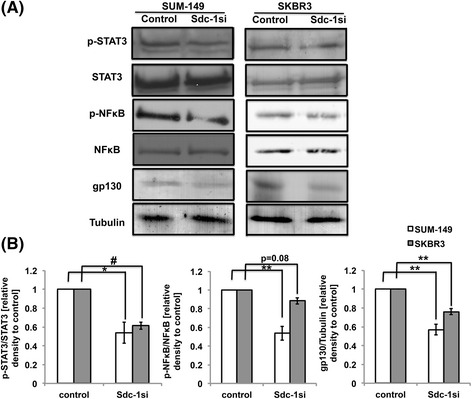
Syndecan-1 silencing attenuates the activation of STAT-3 and NFκB signaling pathways and downregulates protein expression of gp130 in SUM-149 and SKBR3 cells. Seventy-two hours post transfection total cell lysates of control and Syndecan-1 knockdown cells were collected, electrophoresed and immunoblotted. The membrane was probed with the indicated antibodies. **a** Western blot showing expression of p-STAT-3, STAT-3, p-NFκB, NFκB and gp130 upon Syndecan-1 silencing in SUM-149 and SKBR-3 cells. **b** Immunoblot band intensities were normalized to the total form of STAT-3, NFκB or tubulin expression. Data shown are a single experiment representative of three independent experiments. * *P* < 0.05, ** *P* < 0.01 and # *P* < 0.001 as determined by Student’s t-test

We have previously shown that Syndecan-1 modulates the expression of IL-6, IL-6R in different experimental models of inflammation and in MDA-MB-231 breast cancer cells [16, 49, 50]. As IL-6 and its IL-6R/gp130 receptor complex mediate breast CSC self-renewal via STAT3 activation [27], we investigated whether Syndecan-1 depletion might affect expression of gp130 and the active status of STAT3 using Western blot analysis. Relative to controls, gp130 was significantly downregulated at the protein level by 43% and 24% in Syndecan-1 siRNA transfected SUM-149 and SKBR3 cells, respectively (Fig. 6a&b). Interestingly, Syndecan-1 depletion significantly attenuated the active phosphorylated form of STAT3 by 46% and 39% in SUM-149 and SKBR3 cells, compared to control cells, respectively (Fig. 6a&b). The transcription factor NFκB is a master regulator of a number of cytokines (e.g. IL-6 and IL-8) involved in stemness regulation in the triple negative breast cancer [51]. The level of the phosphorylated form of NFκB was downregulated by 46% in SUM-149 (Fig. 6a&b). In contrast, the phosphorylated form of NFκB was downregulated by only 12% in SKBR3 cells upon Syndecan-1 depletion compared to controls, and it did not reach the significance level (*p* = 0.08) (Fig. 6a&b).

### Notch-1 and -3 are positively correlated with Syndecan-1 mRNA expression in tissues of triple negative IBC vs non-IBC

**Fig. 7:**
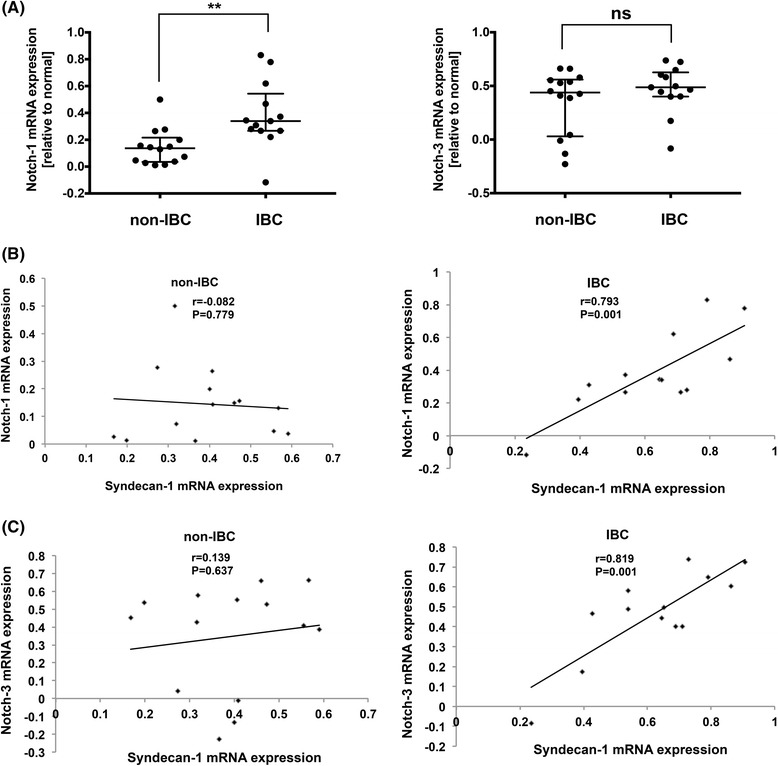
Expression of Notch-1 & -3 transcript levels and their correlation with Syndecan-1 mRNA expression in clinical samples of IBC vs non-IBC patients. **a** Scatter plot shows a significant upregulation of Notch-1 mRNA (*left panel*) but not Notch-3 mRNA levels (*right panel*) in tissues of IBC (*n* = 13) vs non-IBC (*n* = 14). RQ values of mRNA expression are log2-transformed and normalized to values of normal tissues collected during reduction mammoplasty. *Bars* represent median with interquartile range. ** *P* < 0.01 as determined by Mann-Whitney U-test. **b** Pearson’s correlation between Syndecan-1 and Notch-1 mRNA expression in tissues of in non-IBC (*left panel*) and IBC (*right panel*) **c** Pearson’s correlation between Syndecan-1 and Notch-3 mRNA expression in tissues of non-IBC (*left panel*) and IBC (*right panel*)

We next assessed expression of Notch-1 & -3 transcript levels in tissues of IBC vs non-IBC. qPCR results revealed that Notch-1 was significantly upregulated in IBC in comparison with non-IBC (*P* < 0.01), whereas we couldn’t detect a significant difference for expression of Notch-3 mRNA in IBC vs non-IBC (Fig. 7a). Interestingly, a significant positive correlation between Notch-1 and Syndecan-1 mRNA levels (*r* = 0.793, *P* = 0.001) (Fig. 7b) and between Notch-3 and Syndecan-1 mRNA levels (*r* = 0.819, *P* = 0.001) (Fig. 7c) did exist in carcinoma tissues of IBC. This correlation was not observed in non-IBC (Fig. 7b&c).

### Syndecan-1 orchestrates colony formation and expression of inflammatory cytokines via Notch signaling in SUM-149 cells

**Fig. 8:**
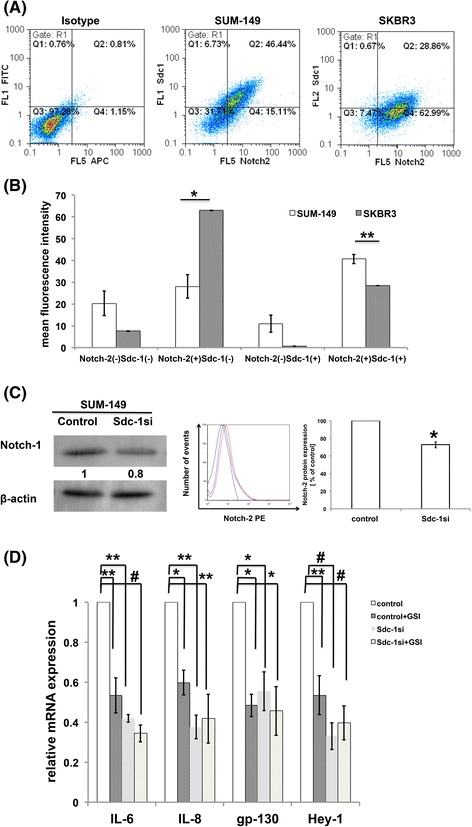
Syndecan-1 regulates expression of Notch-1 &-2 in SUM-149 cells. **a** A representative flow cytometric analysis for the expression of Syndecan-1 and Notch-2 in SUM-149 and SKBR-3. **b** Quantitative analysis of four subpopulations; Notch-2^(-)^Syndecan-1^(-)^, Notch-2^(+)^Syndecan-1^(-)^, Notch-2^(-)^Syndecan-1^(+)^ and Notch-2^(+)^Syndecan-1^(+)^. Data represent mean ± SEM, n ≥ 3. * *P* < 0.05, ** *P* < 0.01 as determined by Student’s t-test. **c** Syndecan-1 knockdown downregulates expression level of Notch-1 protein analyzed by Western blot (*left panel*) and Notch-2 protein analyzed by flow cytometry (*right panel*) in SUM-149 cells. *Black line*: unstained cells, *red line*: control cells, *blue line*: Syndecan-1 knockdown cells. * *P* < 0.05 as determined by Student’s t-test. Data shown are a single experiment representative of three independent experiments. **d** IL-6, IL-8, gp130 and Hey-1 mRNA levels post GSI treatment in SUM-149 cells. Data represent mean ± SEM, n ≥ 3. * *P* < 0.05, ** *P* < 0.01 and ^#^ *P* < 0.001 as determined by one-way ANOVA followed by Tukey’s multiple comparison test

It has been shown that treatment of patient-derived xenograft tumors with anti-Notch-2 antibodies inhibits tumor growth and reduces the tumor-initiating cell frequency [39], suggesting the role played by Notch-2 in regulating CSC properties. Thus, we performed flow cytometric analysis for the expression and distribution of Notch-2 and Syndecan-1 in SUM-149 and SKBR3 cells. Our findings indicate that the Notch-2^(+)^Syndecan-1^(+)^ subset represents approximately 40.7% and 28.5% in SUM-149 and SKBR-3 cells, respectively. The Notch-2^(+)^Syndecan-1^(-)^ subset represents 28.1% and 63.1%, and the Notch-2^(-)^Syndecan-1^(+)^ subset represents 10.95% and 0.72% in SUM-149 and SKBR-3 cells, respectively (Fig. 8a&b). This means that the Notch-2^(+)^-enriched Syndecan-1 subset constitutes 78.8% and 97.5% of total Syndecan-1 expression in SUM-149 and SKBR-3 cells, respectively. These data along with our findings in clinical samples highlights a functional link of Syndecan-1 expression to Notch signaling. Therefore, we tested whether Syndecan-1 is implicated in regulation of Notch expression. Our Western blot data indicate that Syndecan-1 knockdown cells exhibited a significant reduction of Notch-1 full-length protein levels by 20% compared to control cells (Fig. 8c). Furthermore, we sought to evaluate the impact of Syndecan-1 silencing on Notch-2 expression in SUM-149 cells. Our flow cytometric analysis demonstrated a significant downregulation of Notch-2 expression by 27% in Syndecan-1-silenced SUM-149 relative to control cells (*P* < 0.05) (Fig. 8c).

As we have shown that Syndecan-1 knockdown reduced expression of IL-6 and IL-8, the predominant cytokines implicated in IBC stemness regulation, we evaluated whether this effect is Notch-dependent. Therefore, control and Syndecan-1-silenced SUM-149 cells were incubated with 1 μM GSI for 24 h. qPCR results uncovered that Notch inhibition or Syndecan-1 silencing significantly downregulated expression of IL-6, gp130, IL-8 and Hey-1 by at least 40% as compared to control cells (Fig. 8d). Of note, we did not observe any significant additive effect for Notch inhibition in Syndecan-1-silenced cells, suggesting that Syndecan-1 knockdown and Notch exert their potent effect via the same downstream target. To further prove that Syndecan-1 silencing has a functionally similar effect of Notch inhibition, we tested the effect of 1 μM GSI on colony formation. Our data indicate that treatment with GSI abrogated completely colony formation in control and Syndecan-1 siRNA transfected SUM-149 cells (Data not shown).

### Syndecan-1 regulates EGFR expression via Notch signaling and promotes EGF-induced colony formation in IBC

**Fig. 9:**
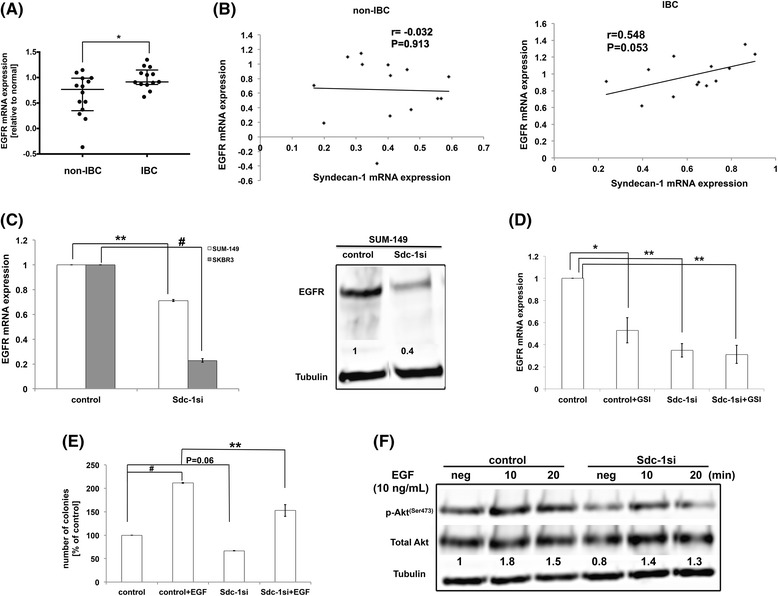
Syndecan-1 is a modulator of EGFR expression and activation via Notch signaling in IBC. **a** Scatter plot shows a significant upregulation of EGFR mRNA in tissues of IBC (*n* = 13) vs non-IBC (*n* = 14). RQ values of EGFR mRNA expression in tissues of IBC vs non-IBC are log2-transformed and normalized to values of normal tissues collected during reduction mammoplasty. *Bars* represent median with interquartile range. * *P* < 0.05 as determined by Mann-Whitney U-test. **b** Pearson’s correlation between Syndecan-1 and EGFR mRNA expression in tissues of non-IBC tissues (*left panel*) and IBC (*right panel*). **c** EGFR mRNA and protein level expression in control and Syndecan-1-silenced SUM-149 and SKBR3 cells. ** *P* < 0.01 and ^#^ *P* < 0.001 as determined by Student’s t-test. **d** Expression of EGFR mRNA levels in control and Syndecan-1-silenced SUM-149 cells post GSI treatment. *P < 0.05 and ** *P* < 0.01 as determined by one-way ANOVA followed by Tukey’s multiple comparison test. **e** Colony formation post 10 ng/ml EGF treatment in control and Syndecan-1-silenced SUM-149 cells. ** *P* < 0.01 and ^#^ *P* < 0.001 as determined by one-way ANOVA followed by Tukey’s multiple comparison test. **f** Western blot analysis of the downstream signaling p-Akt^(Ser473)^ of EGFR signaling in response to EGF stimulation in control and Syndecan-1-silenced SUM-149 cells. Data represent mean ± SEM, n ≥ 3. Data shown are a single experiment representative of three independent experiments

EGFR plays an essential role in IBC progression [31] and is correlated with Syndecan-1 expression in some tumor entities [36, 52]. However, the correlation between Syndecan-1 and EGFR in IBC is still unexplored. Therefore, we sought to evaluate expression of EGFR mRNA and establish a correlation with Syndecan-1 mRNA in triple negative IBC vs non-IBC. Our qPCR data demonstrate EGFR mRNA was significantly higher expressed in tissues of IBC in comparison to those of non-IBC (*P* < 0.05) (Fig. 9a). Interestingly, we found a significant positive correlation between Syndecan-1 and EGFR mRNA expression in tissues of IBC (*r* = 0.548, *P* = 0.05) (Fig. 9b), although this correlation was not observed in tissue of non-IBC (*r* = -0.032, *P* = 0.913) (Fig. 9b). These data prompted us to investigate the effect of Syndecan-1 depletion on EGFR expression in SUM-149 and SKBR3 cells. Our findings indicate that Syndecan-1 knockdown significantly reduced the mRNA expression level of EGFR by 29% in SUM-149 (*P* < 0.01) and by 77% in SKBR3 cells (*P* < 0.001) (Fig. 9c). Relative to control, we validated downregulation of EGFR protein expression by 60% upon Syndecan-1 knockdown in SUM-149 cells as determined by Western blot (Fig. 9c).

Since EGFR is known to be in a crosstalk with Notch signaling in different tumor entities including triple negative breast cancer [53, 54], we examined the effect of GSI on expression of EGFR transcript levels in control and Syndecan-1-depleted SUM-149 cells. Our qPCR data indicate a significant downregulation of EGFR mRNA level by 47% (*P* < 0.05) in control cells upon Notch inhibitor treatment (Fig. 9d). Strikingly, treatment of Syndecan-1-silenced cells with Notch inhibitor did not further decrease EGFR expression, suggesting that EGFR expression is regulated by Syndecan-1 and Notch signaling. This finding was also substantiated at the functional level by treatment of control and Syndecan-1 knockdown SUM-149 cells with EGF to test its effect on colony formation. Syndecan-1 silencing significantly reduced colony formation in response to EGF by 58% (*P* < 0.01) relative to EGF-treated control cells (Fig. 9e). Finally, we studied the effect of Syndecan-1 knockdown on the cell survival downstream target Akt of EGF/EGFR signaling in SUM-149 cells. As depicted in Fig. 9f, our Western blot data demonstrate Syndecan-1 silencing did not only downregulate basal level of the active form of Akt by 20% but also attenuated its activation status in response to EGF by 22% and 10% after 10 and 20 min stimulation compared to control SUM-149 cells, respectively.

## Discussion

As Syndecan-1 is an important modulator of inflammation and the CSC phenotype in different experimental models and in cancer [19, 20], it emerges as a candidate marker for IBC. The current study demonstrates for the first time a higher transcript levels and immunohistochemical staining of Syndecan-1 in clinical samples of triple negative IBC vs non-IBC patients. This is consistent with the prognostic value of Syndecan-1 in different cancer entities, including breast cancer [55] and in line with the negative correlation between the ER, PR and the proportion of CD138-positive cells in ductal breast carcinoma in situ [23]. Interestingly, a higher expression of CD44 with a positive correlation with Syndecan-1 exists in tissues of IBC patients. Of note, Syndecan-1 expression is enriched in CD44^+^ subpopulation in SUM-149 cells, although this enrichment is less in SKBR3 cells. This is in agreement with the notion of interaction between Syndecan-1 and CD44 promoting glioma cell invasion [56] and suggesting a physical and functional association as previously described [57, 58].

To extend our findings to in vitro models and to better understand its functional role, we studied the impact of Syndecan-1 silencing on CSC properties, namely ALDH1 activity and the presence and size of the CD44^(+)^CD24^(-/low)^ subpopulation, in SUM-149 cells. Our data revealed that Syndecan-1 silencing diminished the CD44^(+)^CD24^(-/low)^ and ALDH1-positive subsets compared with controls. These results are consistent with our previous data and other reports demonstrating that Syndecan-1 acts as a regulator of CSCs in triple-negative and ER-positive breast cancer [16, 29] and in prostate cancer [43]. These findings were confirmed in SKBR3 cells. ALDH1 positive cells were reduced upon Syndecan-1 silencing in this cell line. Taken together, these data provide evidence for a role of Syndecan-1 as a regulator of a CSC phenotype in different molecular subtypes of IBC and non-IBC cell lines.

One of the characteristic features of CSCs is the ability to form spheroids and colonies [16, 59]. Our in vitro colony and 3D spheroids formation assays revealed decreased numbers of spheroids formed in 3D and a reduction of colony numbers upon Syndecan-1 knockdown in SUM-149 and SKBR3 cells. This finding is supported by different reports: we have previously shown reduced mammosphere formation and impaired differentiation into cysts in Syndecan-1-depleted MCF-7 cells [16]. Another study showed that early intervention with a Syndecan-1 inhibitor (OGT2115) or RNAi-mediated Syndecan-1 silencing in a transgenic mouse model of prostate cancer reduced the incidence of adenocarcinoma and the number of c-kit^(+)^/CD44^(+)^ cells in cancer foci [43].

It is well-known that breast CSCs are substantially regulated by a multitude of signaling pathways, including the IL-6/STAT3, Notch and Hedgehog pathways, and that targeting these pathways represents potential therapeutic approaches [28]. In this regard, we explored in this study the role of Syndecan-1 in regulating expression of components of the Notch signaling pathway. Interestingly, we found a higher expression of Notch-1 mRNA and a significant positive correlation between Notch-1 & -3 and Syndecan-1 mRNA levels in carcinoma tissues of triple negative IBC vs non-IBC. Moreover, Syndecan-1 is expressed in a Notch-2^(+)^-enriched subset with a prominent higher proportion in SUM-149 than that in SKBR3 cells. Additionally, our findings revealed that Syndecan-1 depletion led to downregulation of Notch-1, -3 and -4, and the Notch signaling downstream target Hey-1 at the mRNA levels, and of Notch-1 & -2 at the protein levels in SUM-149 cells. In contrast, only the mRNA level of Notch-3 was reduced in SKBR3 cells upon Syndecan-1 silencing. In support of our data, it was reported that the neural stem cells expressing both Syndecan-1 and Notch-1 have a higher capacity to form neurospheres than singly positive cells [60]. Another study demonstrated the presence of reciprocal regulation between Notch-2 & -3 and Syndecan-2 in vascular muscle cells with a physical interaction between Syndecan-2 and Notch-3 [61]. Although Notch-2 has a dual role as a tumor suppressor or oncogene in breast cancer (reviewed in [62]), a recent study showed that treatment of patient-derived xenografts of epithelial tumors including breast with the Notch-2/Notch-3 antagonist tarextumab suppressed tumor growth and reduced tumor-initiating cell frequency [39]. In light of this finding, this is the first study reporting that Notch expression is influenced by Syndecan-1 in IBC.

IBC is known to secrete angiogenic and also vasculogenic growth factors, such as VEGF, bFGF, IL-6, and IL-8 [63]. Coordinate expression and secretion of IL-6, IL-8 and GRO-α via NFκB promote tumorgenesis and are associated with poor outcome in triple negative breast cancer patients [51]. GRO chemokines are reported to enhance breast cancer metastasis and resistance to chemotherapy [64]. The maintenance of breast CSCs and their chemoresistance particularly in the basal subtype/triple negative breast cancer is essentially attributed to the synergistic effect between IL-6 [27, 65] and IL-8 [66, 67]. Moreover, IL-6 promotes breast cancer bone metastasis through Notch-1 [68], and induces mammosphere formation in breast cancer cells through Notch-3 [65]. These data thus integrate the IL-6/STAT3 and Notch signaling pathways with relevance to our findings in IBC. SUM-149 cells secrete detectable levels of IL-6 and IL-8, and their expression enhances mammosphere formation and protects SUM-149 cells from radiation upon treatment with the Notch inhibitor RO4929097 [41]. We suggest that this effect can be dampened by Syndecan-1 downregulation. Indeed, treatment of SUM-149 cells with Notch inhibitor reduced expression of IL-6, IL-8 and gp130 mRNA levels to the same extent as Syndecan-1 knockdown without any additive effect of Notch inhibitor in Syndecan-1-depleted cells. Strikingly, the same effect was also observed for the direct downstream Notch target gene Hey-1, suggesting that Syndecan-1 and Notch signaling converge on the same downstream target. However, a potential caveat is associated with the interpretation of the gamma-secretase inhibitor study: Pasqualon et al. [69] have recently shown in a lung cancer model that the transmembrane fragment generated by Syndecan-1 shedding undergoes intramembrane proteolysis by gamma-secretase. If similar mechanisms apply to IBC cells, gamma-secretase inhibitor treatment may not only have directly affected the Notch signaling pathway, but also signaling events triggered by release of the cytoplasmic cleavage fragment of Syndecan-1 [70]. Overall, our data suggest the existence of a signaling axis involving Syndecan-1, Notch, IL-6/gp130 and IL-8 in IBC. Depletion of Syndecan-1 did not only downregulate expression of IL-6 and IL-8 but also their secretion, thus inhibiting the positive autocrine feedback loop.

There is mounting evidence that the expression of inflammatory cytokines including IL-6 is regulated by the transcription factors NFκB and STAT3 [71]. In fact, the NFκB transcription factor pathway contributes to the phenotype of IBC and its target genes are elevated in ER- versus ER+ breast tumors [72]. IL-6 is a direct regulator of breast CSC self-renewal [65] and IL-6/JAK2/STAT3 pathway is more active in CD44^(+)^CD24^(-/low)^ breast cancer cells compared with other tumor cell types and its inhibition blocks the growth of xenografts [27]. A constitutively active STAT3 status is found in about 50–60% of breast tumors specifically in IBC after neoadjuvant chemotherapy [73], which is associated with tumorigenesis and drug resistance [74]. Moreover, STAT3 inhibition represses CSC traits in HER2-positive breast cancers [74]. In this context and in agreement with our prior observation in the triple negative MDA-MB-23 l cells [16], Syndecan-1 knockdown reduced the levels of the activated forms of NFκB and/or STAT3 and downregulated expression of the IL-6/LIF coreceptor gp130 in SUM-149 and SKBR3 cells. Our findings in SKBR3 cells are supported by the observation of an increased IL-6 expression upon HER-2 overexpression, which leads to enhanced breast CSC activity and resistance against anti-HER2 treatment via a STAT3/Akt/NFκB signaling-mediated autocrine-positive feedback loop [75, 76]. Taken together, this proves the efficacy of Syndecan-1 targeting in dampening the inflammatory signaling mediated by NFκB or STAT3 in the two cellular models of different breast cancer subtypes.

An important cue for IBC pathogenesis and progression is EGFR [34]. Our data suggest presence of cross-talk between EGFR and Syndecan-1 in IBC. This is reflected by downregulation of EGFR mRNA and protein levels in SUM-149 and the positive correlation in the clinical samples of IBC. Interestingly, we demonstrated that Notch inhibition did not further downregulate expression of EGFR in Syndecan-1-silenced cells, suggesting that Syndecan-1 regulates expression of EGFR via Notch signaling. This is in agreement with the notion of the crosstalk of EGFR with Notch signaling in triple negative breast cancer and their dual inhibition drastically attenuated active Akt^(Ser473 )^ [53, 77]. Given the coreceptor function of Syndecan-1 for growth factors [18] and downregulation of EGFR expression upon Syndecan-1 silencing, we found downregulation of the EGFR downstream signaling cue pAkt^(Ser473 )^ upon treatment with EGF in Syndecan-1 knockdown cells compared to control SUM-149 cells. At the functional level, Syndecan-1 silencing reduced EGF-induced colony formation compared to control SUM-149 cells. Taken together, our results suggest that Syndecan-1 further regulates a CSC phenotype via EGFR expression and implies a role of interconnected Syndecan-1, Notch and EGFR signaling in IBC.

## Conclusions

**Fig. 10:**
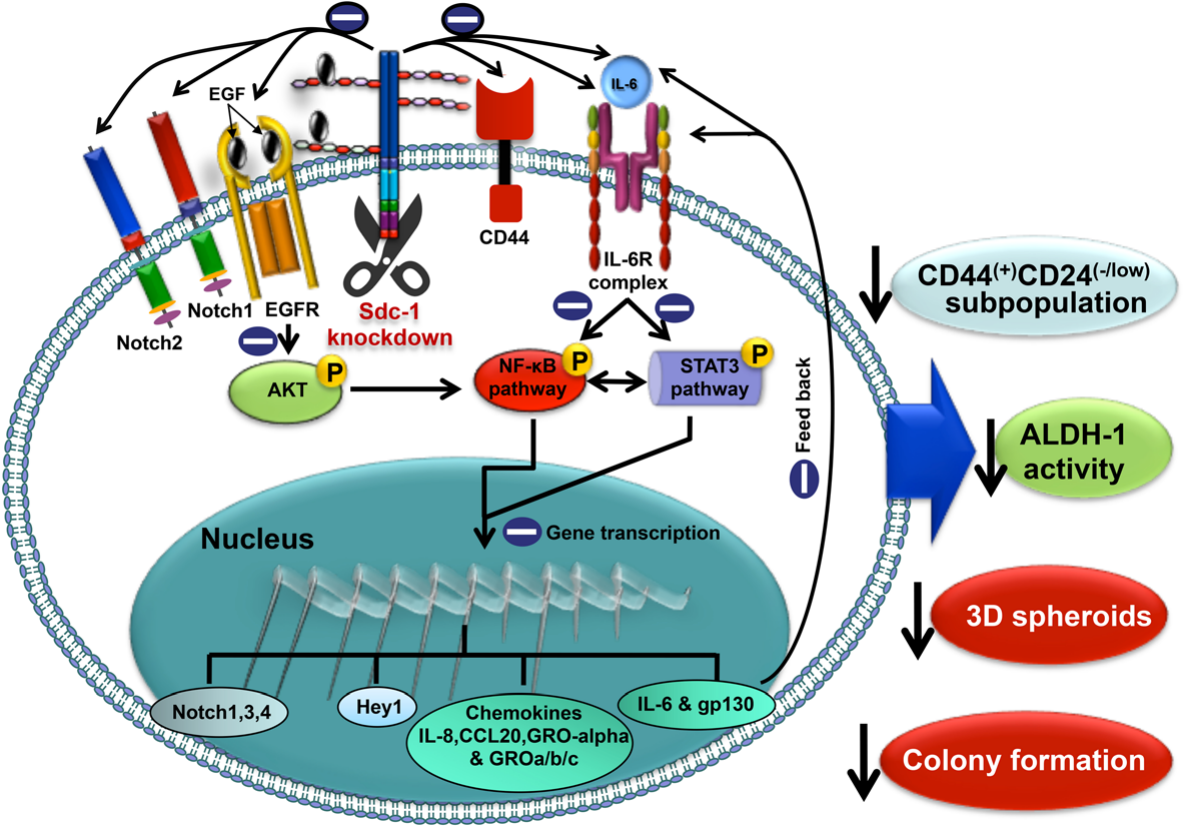
Summary of the mode of action exerted by Syndecan-1 in IBC progression. Syndecan-1 modulates expression and activation of the components of multiple signaling pathways including Notch, EGF/EGFR, and IL-6/STAT3/gp130. These changes have an impact on several features of the breast CSC phenotype, including CSC marker expression, and the formation of colonies and 3D spheroids

In conclusion, this study identifies Syndecan-1 as a novel molecular marker in IBC patients and future studies on larger patient collectives will help to define the full prognostic and predictive value of Syndecan-1 in IBC. Additionally, our data provide evidence for the role played by Syndecan-1 in synchronously fine tuning multiple signaling pathways including IL-6/STAT3/gp130, inflammatory cytokines, Notch, and EGFR, implicated in breast cancer stemness (Fig. 10). Therefore, this study underscores the translational relevance of Syndecan-1 targeting to dampen multiple and intersected signaling pathways-induced CSC phenotype in triple negative IBC patients.

## Additional files

Additional file 1: Table S1. Primers sequences. (DOCX 18 kb)
Additional file 2: Figure S1. A representative image of tumor emboli, a unique feature for tissues of IBC patients showing a positive staining for CD44 and Syndecan-1. (ZIP 2337 kb)
Additional file 3: Figure S2. Flow cytometric analysis of Syndecan-1 expression in control and Syndecan-1 siRNA transfected SUM-149 cells. 500,000 cells were stained for isotype control mouse IgG1-PE and mouse anti-human Syndecan-1 (CD138)-PE and the cells were subjected to flow cytometry. Each plot shows mouse IgG-PE control (dotted line) and CD138-PE-stained cells (solid line). The median fluorescence intensity (MFI) of events is given for each peak. Data are a single experiment representative of three independent experiments. (ZIP 281 kb)

## Abbreviations

ALDH-1: Aldehyde dehydrogenase-1
ANOVA: Analysis of variance
CSC: Cancer stem cell
DEAB: Diethylaminobenzaldehyde
EGFR: Epidermal growth factor receptor
ER/PR: Estrogen receptor/Progesterone receptor
FCS: Fetal calf serum
GSI: Gamma secretase inhibitor
HER-2: Human epidermal growth factor receptor
IBC: Inflammatory breast cancer
NFκB: Nuclear factor kappa B
qPCR: Quantitative real time polymerase chain reaction
Sdc-1: Syndecan-1
siRNA: Small interfering ribonucleic acid
STAT3: Signal transducer and activator of transcription 3
