# Supplementary material for: Syndecan-1 is a novel molecular marker for triple negative inflammatory breast cancer and modulates the cancer stem cell phenotype via the IL-6/STAT3, Notch and EGFR signaling pathways
Source: Mol Cancer. 2017 Mar 7;16:57. doi: 10.1186/s12943-017-0621-z (PMC5341174; doi:10.1186/s12943-017-0621-z)
Supplement: Additional file 1: Table S1. — Primers sequences. (DOCX 18 kb) [file 12943_2017_621_MOESM1_ESM.docx]

**Additional file 1: Table S1 Primers sequences.**

| **Gene** | **Primer sequences/primer code** | |
| --- | --- | --- |
|  | **Forward primer** | **Reverse primer** |
| 18S rRNA | AACCCGTTGAACCCCATT | CCATCCAATCGGTAGTAGCG |
| Notch-1 | GGTGAGACCTGCCTGAATG | GTTGGGGTCCTGGCATC |
| Notch-3 | TGTGCAAATGGAGGTCGTT | CCTGAGTGACAGGGGTCCT |
| Notch-4 | CACGTGAACCCATGTGAGTC | CACAGTGGAATCCTCCAGGT |
| Hey-1 | GAGATCCTGCAGATGACCGT | AGATAACGCGCAACTTCTGC |
| IL-6 | AGTGAGGAACAAGCCAGAGC | CATTTGTGGTTGGGTCAGG |
| IL-6R | GGGACCATGGAGTGGTAGC | ACTGGTCAGCACGCCTCT |
| gp130 | CGGACAGCTTGAACAGAATGT | ACCATCCCACTCACACCTCA |
| IL-8 | CAAGAGCCAGGAAGAAACCA | AGCACTCCTTGGCAAAACTG |
| CCL20 | GTGCTGCTACTCCACCTCTG | CGTGTGAAGCCCACAATAAA |
| Gli1 | AGCGTGAGCCTGAATCTGTG | AGCGTGAGCCTGAATCTGTG |
| ALDH1A1 | GCACGCCAGACTTACCTGTC | CCTCCTCAGTTGCAGGATTAAAG |
| EGFR | CAGCCTACAGTTATGTTCAGTCACACA | GGGCTCTGACTGATCTGGGAGT |
| GAPDH | Hs_GAPDH_1_SG QuantiTect Primer Assay, Cat. no. QT00079247  from Qiagen (Hilden, Germany) | |
