# Supplementary figures and images for: Syndecan-1 is a novel molecular marker for triple negative inflammatory breast cancer and modulates the cancer stem cell phenotype via the IL-6/STAT3, Notch and EGFR signaling pathways
Source: Mol Cancer. 2017 Mar 7;16:57. doi: 10.1186/s12943-017-0621-z (PMC5341174; doi:10.1186/s12943-017-0621-z)

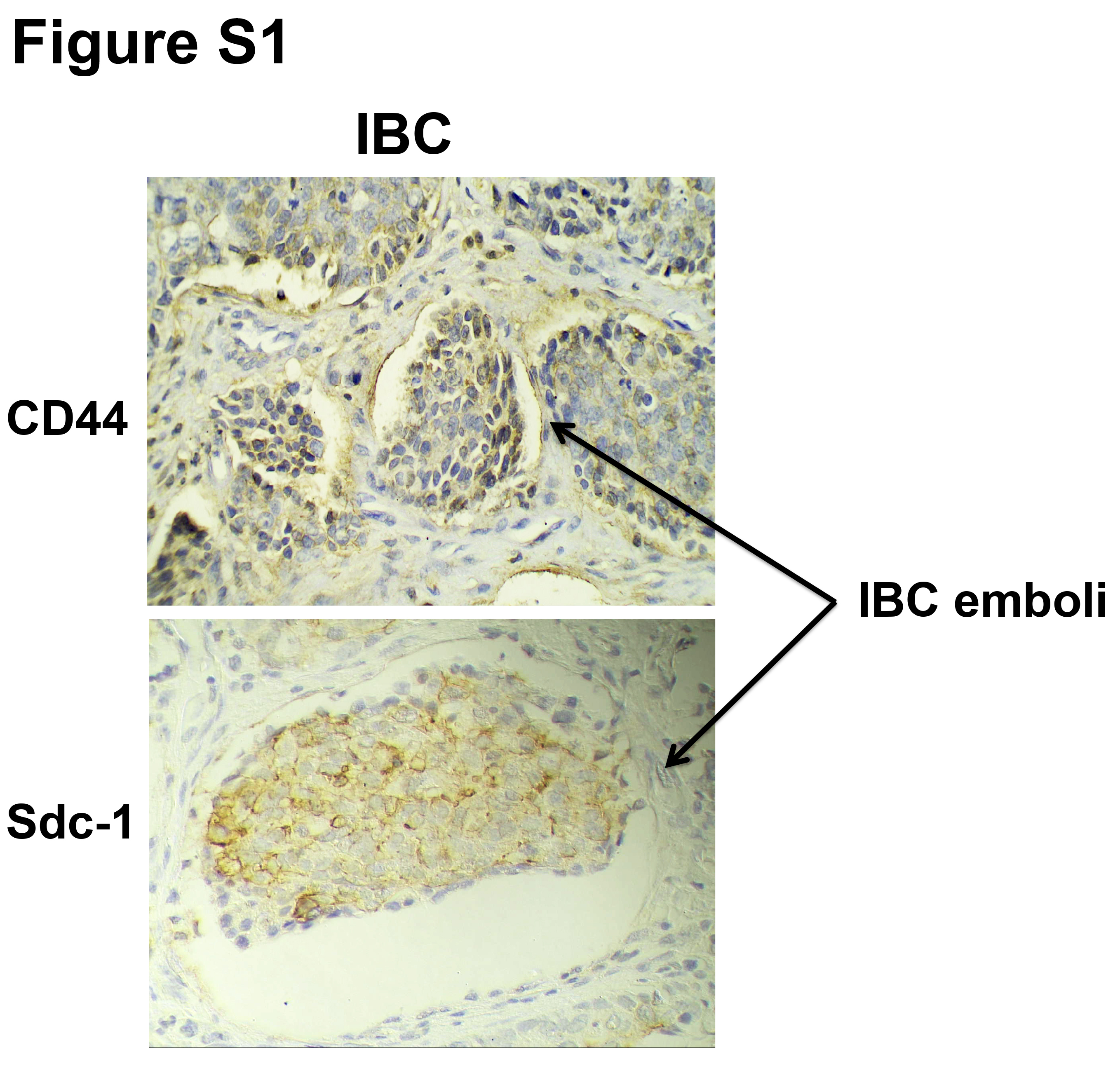

Supplement: Additional file 2: Figure S1. — A representative image of tumor emboli, a unique feature for tissues of IBC patients showing a positive staining for CD44 and Syndecan-1. (ZIP 2337 kb) [file 12943_2017_621_MOESM2_ESM.zip › Figure S1]

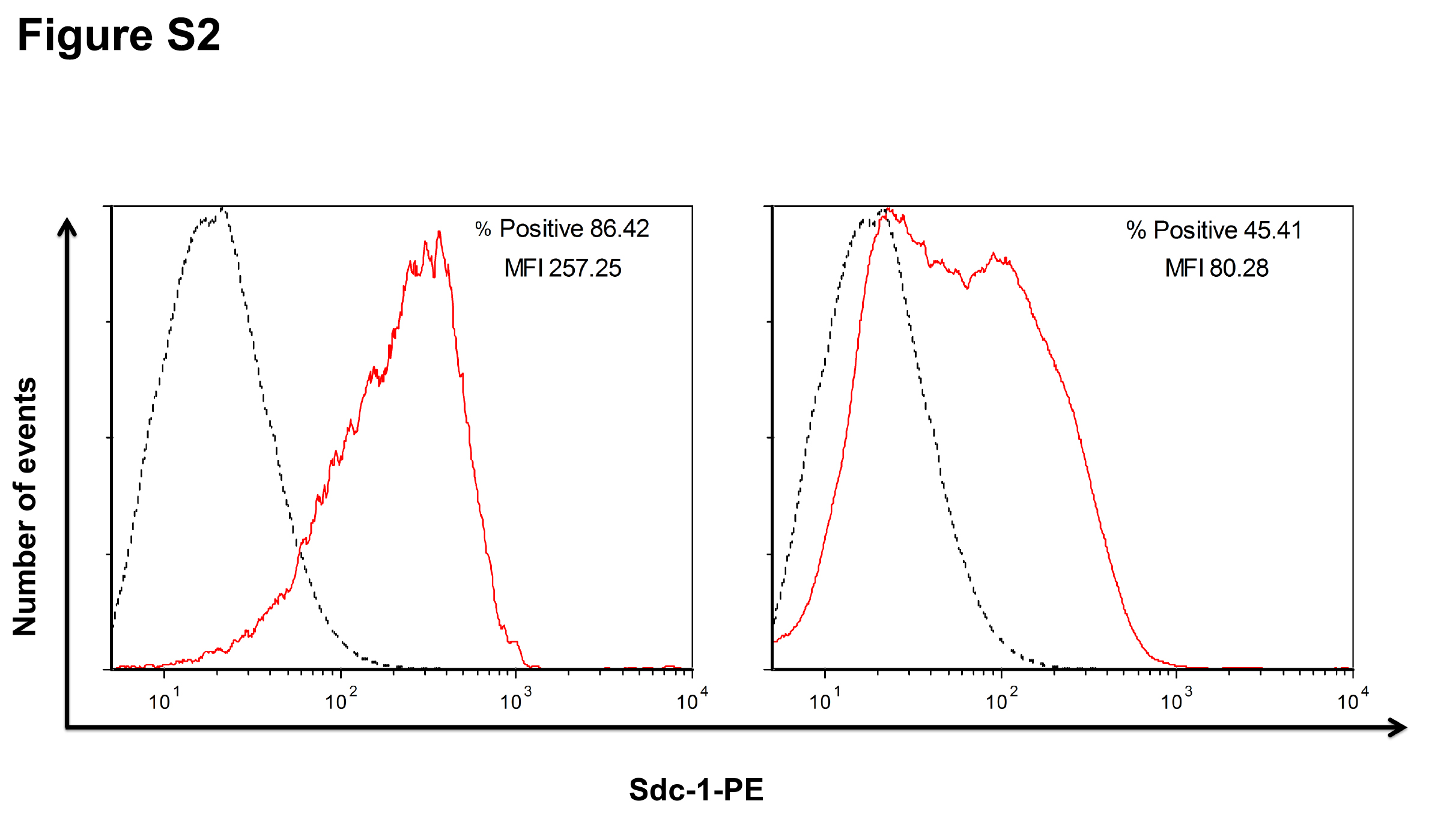

Supplement: Additional file 3: Figure S2. — Flow cytometric analysis of Syndecan-1 expression in control and Syndecan-1 siRNA transfected SUM-149 cells. 500,000 cells were stained for isotype control mouse IgG1-PE and mouse anti-human Syndecan-1 (CD138)-PE and the cells were subjected to flow cytometry. Each plot shows mouse IgG-PE control (dotted line) and CD138-PE-stained cells (solid line). The median fluorescence intensity (MFI) of events is given for each peak. Data are a single experiment representative of three independent experiments. (ZIP 281 kb) [file 12943_2017_621_MOESM3_ESM.zip › Figure S2]
